# Physical Activity and Sleeping Duration Among Adolescents in the US

**DOI:** 10.7759/cureus.29669

**Published:** 2022-09-27

**Authors:** Marc Ganz, Menachem Jacobs, Christopher Alessandro, Samuel Sabzanov, Avrohom Karp, Lulu Wei, Daniel Miller

**Affiliations:** 1 Public Health Sciences, State University of New York Downstate Health Sciences University, New York City, USA; 2 Health Sciences, New York Medical College, Valhalla, USA; 3 Internal Medicine, Icahn School of Medicine, Mount Sinai Queens Hospital Center, New York City, USA

**Keywords:** public health, adolescents, pediatrics, exercise, sleep

## Abstract

Sleep plays a critical role in the development of adolescents. Identifying the factors influencing adolescent sleep duration is a critical public health concern. Our study was designed to look for associations that may affect sleep duration in adolescent students. A cross-sectional research method was used to evaluate a dataset of thousands of adolescent students who were polled. The study assessed whether there was a correlation between exercise and length of sleep. The research showed a statistically significant positive correlation between the studied variables. We found that while demographic variables modify this association, a positive correlation exists even after controlling for these confounders. Our findings suggest that promoting physical activity can increase the quantity of sleep among adolescents.

## Introduction

Sleep has been shown to be vital in the influence and development of physical characteristics such as height and muscle mass, as well as regulating important hormonal changes. Additionally, sleep seems to play an important role in the development of adolescent brains. One study found that synaptic spine elimination of hypocretin and synaptic renormalization was higher in sleeping adolescents than in adult mice, suggesting that sleep deprivation may interrupt the normal process of sleep-related synaptic pruning, highlighting the importance of sleep in the brains of adolescents [1]. Sleep in adolescents may also be related to brain matter volume, as one study found a positive correlation between bilateral hippocampal gray matter volume in children and adolescents who reported high levels of sleep [2]. Sleep seems to be implicated in levels of brain plasticity [3], synaptic homeostasis [4], and the recuperation of neurons [5]. 

Sleep seems to be vital in the psychological and cognitive health of teenagers. Increased levels of sleep are correlated with higher levels of emotional processing [6], cognitive abilities [7], and the consolidation of emotional memories [8]. Sleep seems to be highly correlated to improvements in stable measures of intelligence, such as Intelligence quotient (IQ), as measured by traits like sleep spindles obtained using an electroencephalogram (EEG). The increased magnitude was associated with improved cognitive scores in children and adolescents [9,10]. Insufficient sleep in adolescence is a risk factor for various health disorders, including obesity, type 2 diabetes, cardiovascular diseases, and depression [11-14]. Therefore identifying factors influencing sleep duration in adolescents is key to preventing various health concerns in this population.

Exercise has long been associated with better sleep, and evidence is accumulating, pointing to the efficacy of exercise as a nonpharmacologic treatment for improving sleep quality and duration in adult populations [15]. However, the association between physical activity and sleep in pediatric populations is less understood. Previous groups, specifically Mendelson et al., have suggested a positive association between adolescents' sleep duration and physical activity. Their group showed that physical activity, defined as 12 weeks of formal exercise training, increased sleep duration in adolescents [16]. Exploring the bidirectional nature of this relationship, Garaulet et al. showed that adolescents who slept less than eight hours per day had more sedentary lifestyles measured by accelerometry and spent significantly more time watching television [17]. The data from these groups support a positive correlation between physical activity and sleep duration. However, Kobel et al., Li et al., and Ogawa et al. have shown no association between physical activity and sleep duration [18-20]. These conflicting conclusions in the literature present a challenge in understanding the relationship between physical activity and sleep.

Given the critical role that sleep duration plays in adolescent development, and the preceding conflict mentioned, we set out to further explore this association using the 2019 national Youth Risk Behavior Survey (YRBS) dataset. We show a robust positive correlation between physical activity and sleep duration. While this relationship is mediated by various confounders such as age, gender, race, obesity, and prior diagnosis of depression, the positive correlation between physical activity and sleep duration persists even after controlling for these mediating factors.

## Materials and methods

We used the 2019 national Youth Risk Behavior Survey (YRBS) dataset. This dataset polls schools across the country to monitor risky behaviors in adolescents, such as sexual contact, dietary habits, and illicit substance use. The Physician’s Journal of Medicine Institutional Review Board reviewed and approved this study. Of 181 schools sampled, 136 schools responded, with a response rate of 75%. Of the 17,025 students sampled, 13,677 questionnaires were usable, with a response rate of 80%. From these usable questionnaires, 13,220 participants (96%) filled out questions pertaining to the performance, or lack thereof, of 60+ min/day of physical activity for five or more days/week. This group was the sample population that we used for our analysis.

The outcome variable used in this study was an average of eight or more hours of sleep on a school night - the minimum figure recommended by the CDC for adolescents. This variable was measured by the response to the question regarding the number of hours slept on an average school night. An answer of eight hours, nine hours, or 10+ hours was coded as Yes = True, and an answer of less than or equal to seven hours was coded as No = False. 

The predictor variable used was the self-reported physical activity of 60+ mins/day for five or more days/week. Physical activity was measured by the question of the past seven days: “How many were you physically active for 60+ mins?”. An answer equal to or less than four days was coded for as No = False, while an answer equal to or greater than five was coded for as Yes = True. These two groups were labeled as physically active (PA) and non-physically active (NPA) groups, respectively. Covariates such as gender, age, obesity, depression, and race were then analyzed. 

Statistical analysis 

The Chi-square test was used to measure the association between the predictor and outcome variable. A p-value of less than 0.05 was statistically significant, with a confidence level of 95%. Multivariate logistical regression was used to analyze the relationship between the activity of 60+ mins for five or more days/week and an average of eight or more hours of sleep on a school night, including age, gender, race, and other characteristics such as obesity and depression. Both crude and adjusted odds ratios (OR) and their confidence intervals were computed. All analyses were performed using RStudio (RStudio, Boston, Massachusetts). 

## Results

Of the 5,625 participants in the PA group, 1,460 responded that they slept an average of eight or more hours on a school night (26%). However, of the 7,595 participants who were in the NPA group, 1,434 (19%) did not report an average of eight or more hours on a school night. This indicates a significant (p<0.01) positive correlation between physical activity and quantity of sleep (Table 1). 

**Table 1:** Odds of getting eight or more hours of sleep as a response to physical activity

Dependent variable - sleep duration of eight or more hours		
Independent variables	Odds ratio	Confidence interval
Physical activity	1.31	1.19 - 1.44
Gender		
Male	1.13	1.04 - 1.25
Race/ethnicity		
Black	0.84	0.66 - 1.07
White	1.03	0.83 - 1.27
Asian	0.65	0.49 - 0.89
Hispanic	0.97	0.78 - 1.22
Other	0.78	0.49 - 1.21
Other characteristics		
Obese	0.91	0.79 - 1.04
Depressed	0.53	0.48 - 0.58
Age, mean (SD)	0.85	0.82 - 0.88

Participants in the PA group had higher odds (50%) of reporting an average of eight or more hours of sleep on a school night (crude OR = 1.50; CI = 1.38 - 1.63; Table 2). However, after adjustment for age, gender, race, obesity, and depression, adjusted OR (AOR) = 1.31, CI = 1.19 - 1.44 (Table 2). This shows that, after accounting for covariates, participants in the PA group had higher odds (31%) of reporting an average of eight or more hours of sleep on a school night. Participants who self-identified as males and those who self-identified as White had higher odds of reporting an average of eight or more hours of sleep on a school night (Table 2). Students who self-identified as Asian Americans and those who self-identified as depressed had a decreased probability of reporting an average of eight or more hours of sleep on a school night (Table 2).

**Table 2:** Characteristics of YRBS 2019 participants YRBS - Youth Risk Behavior Survey

Variable	NOT active for 60 min a day for five or more days a week	Active for 60 min a day for five or more days a week	p-value
Totals	7,595 (56%)	5,625 (42%)	
Sleep more eight or more hours at night	1434 (19%)	1460 (26%)	<0.001
Gender			
Male	3151(42.0%)	3220 (57.8%)	<0.001
Female	4360 (58.0%)	2350 (42.2%)	
Race/ethnicity			
Black	1227 (16.7%)	670 (12.2%)	<0.001
White	3445 (46.9%)	3086 (56.4%)	<0.001
Asian	391 (5.3%)	214 (3.9%)	<0.001
Hispanic	1804 (24.5%)	1145 (20.9)	
Other	106 (1.4%)	92 (1.7%)	0.28
Other Characteristics			
Obese	1125 (16.9%)	604 (11.7%)	<0.001
Depressed	3129 (42.1%)	1650 (29.7%)	<0.001
Age, mean (SD)	16.0 (1.3)	15.9 (1.2)	<0.001

## Discussion

This study examined the associations between physical activity and sleep duration among adolescents in the United States. Our results further support an association between physical activity and sleep duration that is affected by gender, race, and depression. Adolescents who were physically active for 60+ mins/day for five or more days/week, as well as those who self-identified as males, white, or as suffering from depression, had higher odds of reporting an average of eight or more hours of sleep on a school night, These findings are consistent with the results of previous groups [17,20,21]. However, as previously discussed, some literature has produced conflicting data regarding the association between sleep duration and physical activity. Here, we show further support for a positive correlation between sleep duration and physical activity, even after controlling for key demographic factors. Our findings suggest that this correlation is manipulable by race, gender, and mental health status. Given the positive correlation, further research, such as meta-analyses and inquiry into the underlying biological changes behind this association, is warranted.

Additionally, we did not differentiate between the nature and difficulty of the activities. Some adolescents may participate in various sports that are not only different in exertion but also may be significantly different physiologically. Various forms of exercise have been shown to stimulate different levels of hormonal factors, with weight-bearing activities being correlated with high levels of insulin-like growth factor 1 (IGF-1) [22], which has been shown to have a positive influence on sleep and may impact the wakefulness and overall quality of sleep of adolescents [23].

Strikingly, in our results, we discovered that adolescents who self-identified as depressed reported fewer hours slept overall. While this correlation has been previously described [24], there has been little data on attempting to remedy the sleep habits of depressed adolescents using exercise. This may be a potent avenue of analysis in addition to our findings that exercise is positively correlated with sleep in adolescents. There have been many studies that reported that exercise was shown to reduce stress and anxiety and inculcate feelings of well-being [25]. This two-pronged approach may assist vulnerable adolescents struggling with anxiety.

Some important limitations of our study were that although the total number of adolescent sleep hours was inventoried, we did not differentiate between the timeframe of exercise sessions. Adolescents who attend school usually have a few breaks throughout the day that they may use to exercise. Some possible variables to examine would be to establish a time frame regarding their exertions to indicate how many hours until they would then proceed to go to sleep. There has been a significant amount of data on the timeframe of exercise regarding sleep being correlated with various levels of stimulatory hormones that may actually inhibit sleep in the short term [18]. This may be rather problematic as adolescents have been shown to sleep fewer hours than physiologically required for optimal development despite requiring the same number of hours as adults [26].

## Conclusions

We were able to show that a self-reported average of eight or more hours of sleep on a school night was positively correlated with physical activity of 60+ mins/day for five or more days/week. Males and whites have higher odds of self-reporting an average of eight or more hours of sleep on a school night compared to Asian Americans. Students that self-identified as depressed also had lower odds of reporting an average of eight or more hours of sleep on a school night. Our findings suggest that possibly promoting physical activity for 60+ mins/day for five or more days/week can increase the quantity of sleep among adolescents. As previously discussed, further investigation into the roles of gender, race, and mental health status in sleep is warranted to understand better why certain adolescents have lower odds of receiving an adequate amount of sleep for proper development. Efforts from policymakers, community leaders, and public health officials to promote physical activity could play a key role in modulating adolescent sleep quantity.
